# Edible Offal as a Valuable Source of Nutrients in the Diet—A Review

**DOI:** 10.3390/nu16111609

**Published:** 2024-05-24

**Authors:** Agnieszka Latoch, Dariusz Mirosław Stasiak, Patryk Siczek

**Affiliations:** 1 Department of Animal Food Technology, University of Life Sciences in Lublin, 8 Skromna St., 20-704 Lublin, Poland; agnieszka.latoch@up.lublin.pl; 2 Department of Plant Food Technology and Gastronomy, University of Life Sciences in Lublin, 8 Skromna St., 20-704 Lublin, Poland; patryk.siczek@up.lublin.pl

**Keywords:** animal by-products, proteins, fatty acid profile, minerals, vitamin, bioactive substances

## Abstract

The global increase in demand for meat leads to substantial quantities of by-products, including edible offal from both wild and domesticated animals raised for diversified consumption products within an agricultural framework. Information on the nutritional value of offal is scattered and limited. This review aims to synthesize scientific publications on the potential of offal as a source of nutrients and bioactive substances in human diets. The literature review included publications available in ISI Web of Science and Google Scholar published between 2014 and 2024. Findings indicate that edible offal is characterized by a nutrient concentration often surpassing that found in skeletal muscle. This review discusses the yield of edible offal and explores factors influencing human consumption. Selected factors affecting the nutritional value of offal of various animals and the importance of individual nutrients in ensuring the proper functioning of the human body were analyzed. The optimal use of offal in processing and catering can significantly benefit aspects of human life, including diet quality, food security, and conservation of natural resources.

## 1. Introduction

Meat and its products are pivotal in sustaining nutritious human diets due to their high nutrient density [1,2,3]. However, the rising demand for these foods negatively impacts the environment and strains natural resources [4,5,6,7]. The optimal use of raw materials from slaughtered animals can reduce greenhouse gas emissions, food waste, and the phenomenon of global hunger [6,8,9,10], and in the present poses a major challenge for both science and industry. Edible by-products can comprise nearly 40% of an animal’s carcass weight [11,12]. They are usually rich in high-quality proteins, vitamins, micronutrients and macronutrients, fats, bioactive peptides, and more, often in quantities matching or exceeding those in skeletal muscle [13,14,15,16,17]. Despite the substantial nutritional value of offal, its utilization remains limited [8,18]. Although it is used in various dishes and products almost all over the world [19,20,21,22,23,24], several factors (e.g., tradition, culture) limit its consumption [19,20,21,25,26,27]. Residents of highly developed countries hardly consume offal [24], which may be due to preferences determined by the availability of other foods. Promoting nutritional benefits and the uniqueness of dishes (e.g., foie gras, ris de veau), along with environmental considerations, could enhance offal consumption rates [7,10,25,28]. Leveraging edible offal expands the variety of meat products and dishes, boosts economic efficiency, enhances nutritional value, decreases waste, and addresses protein deficiencies in the diets of people in developing countries [14,21,29,30,31].

While the scientific literature is abundant with information on the nutritional value, physicochemical properties, and nutritional use of meat, the availability of information on the nutritional value of offal from livestock [3,8,20,32,33], birds [34,35,36,37], and wildlife is limited [38,39], and the information itself is quite scattered. FAO databases, including the Global Livestock Environmental Assessment Model (GLEAM) database, do not provide up-to-date information on the production of edible offal and the nutritional composition of edible offal [40,41]. Furthermore, the U.S. Department of Agriculture database (2011) [42] offers only a limited overview under “diverse meat and by-products”, with a focus that is generally species-specific and rarely addresses nutritional composition variations due to external and internal factors [32]. Additionally, the methodologies behind data acquisition in most food composition databases are not disclosed, leaving discrepancies between databases unexplained. In addition, most food composition databases do not disclose how the data were obtained. For this reason, the apparent differences between databases cannot be explained [43]. Consequently, directing scientific research towards the nutritional value of offal, alongside its health, environmental, and economic impacts, stands as a relevant and innovative course of action [8]. The generation of new scientific insights can enhance awareness among consumers, producers, and scientists about the advantages of offal utilization [44]. Thus, this study aims to examine, through scientific reports from the past decade, offal’s potential as a nutrient source in the human diet. This review underscores the significance of offal as a nutrient-rich and functional ingredient source. It details the yields of various edible organs and the factors influencing their human consumption. It examines the factors impacting the nutritional value of animal offal and the role of specific nutrients in maintaining bodily health.

## 2. Materials and Methods

**Figure 1:**
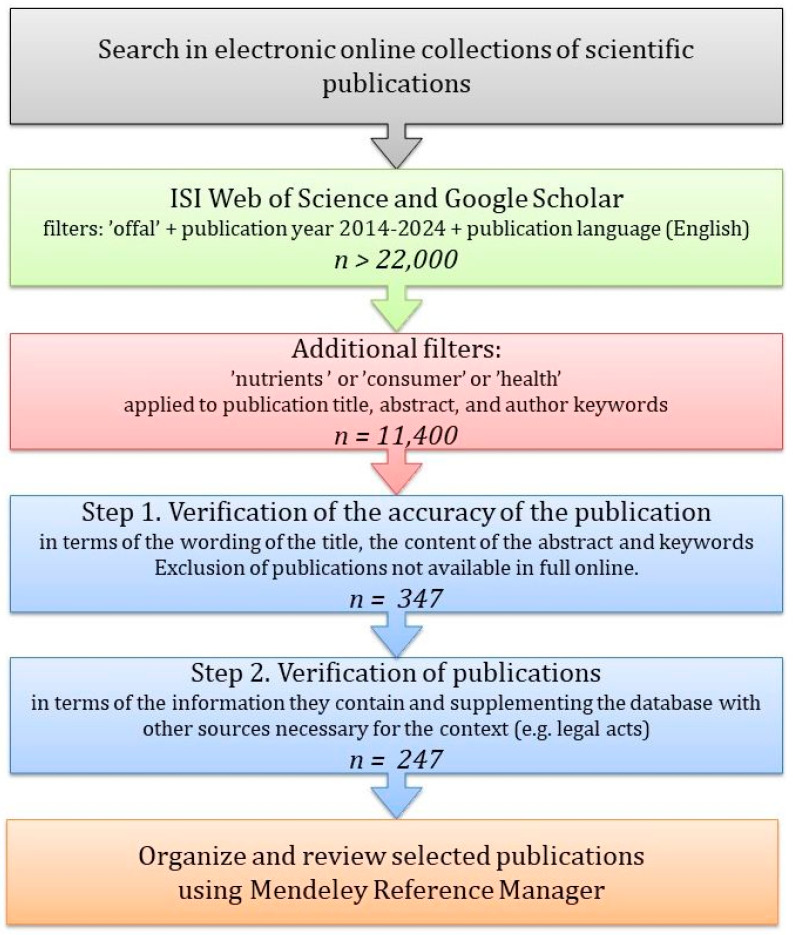
Procedure for searching and selecting publications.

The literature review, conducted in the first quarter of 2024, included publications from the ISI Web of Science and Google Scholar databases published between 2014 and 2024. The initial search utilized “offal” as a keyword, further refined through additional terms such as “nutrients”, “consumers”, and “health” applied to publication titles, abstracts, and author keywords (Figure 1). The literature was organized using the Mendeley Reference Manager application. The review of information was performed in stages, by three researchers. In the first stage, the relevance of publications was verified due to the wording of the title, the content of the abstract, and keywords. Publications inaccessible in full online were excluded. This approach yielded 347 publications for a detailed second-stage review. Based on consensus, the list of publications from 2014 to 2024 was reduced to 247 items classified for this review and supplemented with other information necessary for context. This review amalgamates scientific publications providing information relevant to human nutrition considering, among others, the determinants of yields, chemical composition, and nutritional value of red and white offal in the sense of Reg. (EC) No. 853/2004.

## 3. Definitions of Edible Offal

Animal by-products, also known as the “fifth quarter”, “offal meats”, “organ meats”, or “assortment meats” encompass the nonmeat portions of the carcass [8,29,45,46,47,48] intended for human consumption [49,50,51,52]. The use of animal by-products varies globally, influenced by customs, traditions, culture, and religion [14,53]. The designation of offal as “variety meat” avoids unfavorable associations in consumers related to an edible part of a slaughtered animal other than skeletal muscle [54].

**Figure 2:**
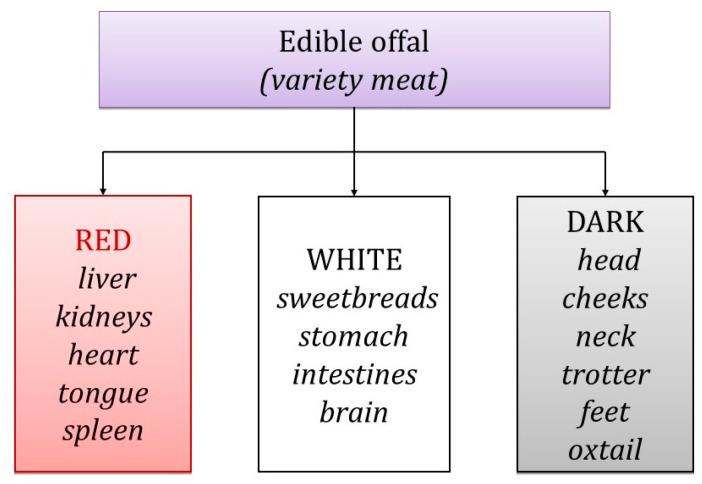
Classification of edible offal within the meaning of Reg. (EC) No. 853/2004.

The European Union (EU) defines [55] “meat” as the edible parts of animals, including blood, whereas “offal” is categorized as fresh meat apart from the carcass, comprising viscera and blood, thus excluding skeletal muscle (Figure 2). Offal encompasses smooth muscle and internal organs like the liver, kidneys, heart, tongue, pancreas, spleen, brain, intestines, lungs [13,19,20,23,29,30,36,39,45,56,57,58,59,60], and blood, all of which serve as food ingredients [4,55].

Offal is classified based on its color. Red offal includes items like the liver, heart, kidney, tongue, spleen, and testicles, which are considered delicacies in some countries [61]. White offal, requiring more extensive heat treatment before consumption, consists of the stomach, lungs, pancreas, thymus, intestines, brain, cheeks, and tail [62,63]. There is also a category known as black offal, which includes parts like the head, shank, neck, and trotter [51,60].

Within the EU, animal by-products are categorized into categories reflecting the health risks they pose to both humans and animals [49]. Categories 1 and 2 comprise animal parts unsuitable for consumption due to their high or medium risk of disease and pathology. Category 3 includes, among others, by-products that are considered low-risk materials that can be used for food production [7].

## 4. Determinants of the Yield of Edible Offal

Edible by-products constitute a significant portion of an animal’s total carcass weight, with offal yields ranging from 10% to 30% depending on various factors such as species, age, sex, live weight, fatness, living conditions, and other factors [3,13,19,24,64,65,66,67,68]. The quality of offal is influenced by both genetic [32] and environmental factors, including the animal’s housing system and nutrition [9,22,69].

In the scientific literature, studies commonly report yields of specific organs like the liver, heart, kidney, tongue, and lungs. There are fewer reports on the yields of other internal organs such as the stomach, spleen, thymus, pancreas, and intestines (Table 1).

The age of an animal significantly modifies the mass proportion of its internal organs. For instance, the organ weights in young cattle are generally 2.5 times less than those in adult animals [14]. The liver consistently ranks as the heaviest organ of the body. In cattle, the liver represents the largest proportion of organ weight (1.0–4.5%), followed by the heart (0.3–0.62%). The weight of calves’ tongues constitutes about 0.45% of their total weight, whereas in adult cattle, this figure is slightly higher at 0.25–0.5%. The kidneys in calves represent 0.45% of their weight, compared to 0.07–0.24% in adult cattle [14]. Similar relationships were not observed between the weight of the internal organs of lambs and rams [3,8].

In pigs, the liver typically weighs around 1.16–2.12 kg, making up 1.0–1.7 of the total slaughter weight, which averages about 100 kg [24,70,71]. Studies show that breed, rearing method, or genotype does not significantly influence the weight and percentage of offal [20,22,69,72,73], with the heart weighing approximately 0.4 kg (0.35%), the kidney 0.34–0.40 kg (0.3%), the tongue 0.25–0.30 kg (0.25%), the lungs 0.67–1.013 kg (0.53–0.77%). Similarly, research on wild animals such as zebras, donkeys, antelopes, wildebeests, and fallow deer indicates no significant seasonal effect on the weight and proportion of internal organs [39,74,75,76,77].

As with cattle [14], the mass proportion of internal organs (liver, kidney, and heart) of wild boars is determined by age and sexual dimorphism [78]. In addition, wild boars, unlike domestic pigs, are characterized by a higher mass proportion of by-products [20,22,59,69,79]. The crossbreeding of domestic pigs and wild boars also impacts the weight and proportion of offal [80]. In wild boars, the liver represents the largest mass share within the carcass (2.55%). Compared to domestic pigs, wild boars have tongues that weigh less and kidneys that weigh more. Despite boars being almost twice as light as pigs, their liver and heart weights are comparable [58].

The largest weight among bird offal, as in the case of mammals, is the liver [36]. The mass range of offal (liver, stomach, and heart) is determined by age, sex, preslaughter weight, and the production system. Notably, organically raised chickens yield a higher proportion of certain pieces of offal (heart, stomach) compared to those raised conventionally [35,81,82]. This difference is attributed to the development of the cardiovascular system in slow-growing chickens [83] and the enhanced stomach weight due to dietary stimulation from foraging on grassy ranges with access to insects, worms, sand, and fiber-rich foods [35,81,82,84].

**Table 1:** Weight (g) of edible offal from various animal species.

Animal and Additional Information	Edible Offal										Reference
	Liver	Kidneys	Heart	Tongue	Lungs	Stomachs	Spleen	Sweetbread	Pancreas	Intestines	
Cattle	983	990	1053	1290	nd	nd	nd	900	nd	nd	[85]
Cattle (Suckler beef) Holstein–Friesian	3094 (1.2) *	587 (0.22) *	1009 (0.37) *	931 (0.35) *	nd	nd	nd	nd	nd	nd	[14]
Calves (Veal calves) Holstein–Friesian	1349 (2.0) *	313 (0.45) *	425 (0.62) *	300 (0.44) *	nd	nd	nd	nd	nd	nd	[14]
Sheep	624	80	187	92	610	2189	109	nd	nd	1837	[8]
Lamb	696	52	192	94	459	1130	72			2009	[8]
Australian lamb	710	75	250	100	450		110	40	110	570	[3]
Pig Polish Great White × Polish Canine; Duroc × Pietrain	1607 (1.41) *	334 (0.30) *	422 (0.37) *	295 (0.26) *	881 (0.77) *	nd	nd	nd	nd	nd	[71]
Pig	1720 (1.72) *	nd	nd	nd	nd	nd	nd	nd	nd	nd	[24]
Pig Landrace	1270 (1.14) *	340	400 (0.36) *	250 (0.23) *	670 (0.60) *	nd	nd	nd	nd	nd	[22]
Pig Pulawska	1410 (1.25) *	380	390 (0.35) *	280 (0.25) *	710 (0.64) *						[22]
Pig Landrace × Yorkshire × Duroc	1820 (1.35) *		440 (0.33) *		710 (0.53) *	750 (0.56) *	220 (0.16) *		240 (0.18) *	Small 1170 (0.86) * Large 1230 (0.90) *	[20]
Pig Pulawska slatted floor rearing system	1160 (1.03) *	340 (0.30) *	390 (0.36) *	250 (0.23) *	670 (0.59) *	nd	nd	nd	nd	nd	[69]
Pig Pulawska deep litter rearing system	1.420 (1.27) *	380 (0.34) *	400 (0.35) *	270 (0.25) *	690 (0.62) *	nd	nd	nd	nd	nd	[69]
Pig—genotype C/T (not susceptible to stress)	1831	400	391	280	997	nd	nd	nd	nd	nd	[59]
Pig—genotype C/C (partially susceptible to stress)	2120	406	407	292	1013	nd	nd	nd	nd	nd	[59]
Zebra (*Equus quagga*) (winter slaughter)	3330 (1.00) *	740 (0.30) *	1790 (0.72) *	nd	3850 (1.20) * with trachea	nd	930 (0.30) *	nd	nd	nd	[39]
Zebra (*Equus quagga*) (summer slaughter)	3320 (1.20) *	648 (0.20) *	1730 (0.53) *	nd	3420 (1.2) * with trachea	nd	970 (0.30) *	nd	nd	nd	[39]
Fallow deer (*Dama dama*) (summer slaughter)	823 (1.97) *	112 (0.27) *	388 (0.93) *	147 (0.35) *	nd	nd	nd	nd	nd	nd	[74]
Fallow deer (*Dama dama*) (winter slaughter)	842 (2.06) *	119 (0.29) *	379 (0.92) *	158 (0.39) *	nd	nd	nd	nd	nd	nd	[74]
Immature wild boar	635 (2.53) *	113 (0.49) *	180 (0.69) *	141 (0.55) *	nd	nd	nd	nd	nd	nd	[78]
Juvenile wild boar	1138 (1.93) *	217 (0.38) *	353 (0.59) *	289 (0.50) *	nd	nd	nd	nd	nd	nd	[78]
Wild boar (*Sus scrofa scrofa*)	1358 (2.55) *	305 (0.57) *	419 (0.79) *	230 (0.43) *	859 (1.62) *	nd	nd	nd	nd	nd	[71]
African ostrich (*Strutio camelus var. domesticus*)	1586	nd	890	nd	nd	1088	nd	nd	nd	nd	[36]
Chicken organic rearing system	44 (1.89) *	nd	13 (0.57) *	nd	nd	37 (1.75) *	nd	nd	nd	nd	[35]
Chicken convectional rearing system	42 (1.85) *	nd	9 (0.39) *	nd	nd	26 (1.16) *	nd	nd	nd	nd	[35]

* Share (% of body weight); nd—no data.

## 5. Consumption of Edible Offal

Consumer preferences and consumption levels of offal are subjects of extensive scientific research [6,7,10,21,30,36,51,56], yet findings remain fragmented. Social, religious, economic, and cultural factors are of great importance in determining consumer behavior in each region [51,60]. Offal is considered a delicacy of nutritional value in some parts of the world; in others, it is a relatively rare food or is considered inedible [19,20,32,33,35,79,86,87]. The nutritional use of offal is relatively unstable and has no small impact on the state of the livestock sector [88]. This affects the range of offal products, which is much smaller compared to the range of meat products [74].

The global consumption of animal by-products shows remarkable diversity [19,21,64] influenced by dietary changes, the rising demand for convenient products [89], health concerns (e.g., BSE) [90], the accumulation of heavy metals (e.g., Cd and Pb) [91], societal and demographic factors [92], dietary neophobia [7,10,31,93], and the availability of raw materials [21]. Although sensory, health, and nutritional properties are primary food selection criteria, economic and cultural aspects (traditions, norms, and customs of a given society) alongside social factors (such as consumer attitudes and beliefs) also play crucial roles in offal consumption decisions [21,31,51,94,95,96].

It is well known that offal tends to have an undesirable image, which can affect consumers’ emotions and behaviors [95]. Food neophobia and food repulsion sensitivity play a crucial role in determining consumers’ willingness to eat them. Food neophobia has been shown to have a direct negative impact on the intention to consume offal [7].

In Europe, a noticeable decline in offal consumption is observed [6,64,97]. For instance, in 2020, Poles consumed approximately 13 g of offal/day, accounting for about 6% of total meat intake and surpassing beef consumption [36,98]. Meanwhile, in Spain, offal consumption has declined to around 0.23 g [6,99,100]. Research by Llauger et al. (2021) [6] indicates that Spaniards value nutritional benefits, environmental concerns (such as reducing food waste and environmental impact), and affordability in their offal purchases. The presence of potentially undesirable compounds (such as toxins or drug residues), appearance and flavour (but not taste) of offal may deter some consumers [101].

Despite its mildly metallic taste, bitter aftertaste, and tenuous texture, which might be unappealing to some, the liver is a staple in many European diets [24]. On average, Europeans consume about 2.47 g of liver/day. Poultry liver is the most consumed type, averaging 0.75 g/day, followed by pork liver at 0.54 g, beef liver at 0.36 g, mutton liver at 0.30 g, veal liver at 0.27 g, goose liver at 0.19 g, and turkey liver at 0.05 g. According to data from the EFSA (2011) [102], the significant volume of liver consumption can be attributed partly to the popularity of pâté [103].

In South Africa, the daily offal consumption averages 13 g/person [38,39,104]. Research by Alao et al. (2018) [21] indicates that the key factors influencing offal purchases include freshness, price, and availability, rather than nutritional value. In Ghana, there is a preference for pork, cattle, and goat livers and stomachs [29,30]. However, in regions with a predominantly Muslim population, traditional preferences lean towards liver, kidney, and heart. Less commonly consumed are the head and feet, stomach, intestines, spleen, and lungs, with the average daily offal consumption per household being about 25 g. Much less frequently consumed are the head and feet, stomach, intestines, spleen, and lungs. Nevertheless, the average daily consumption of offal there is about 25 g per household [51].

In African countries, taste, along with health, economic, cultural, and religious considerations, plays a crucial role in determining the consumption of offal from wild animals. This is because people prefer familiar tastes and often express negative preferences for unknown foods [6,56,105,106].

Offal has an important place in Turkish cuisine. However, as the research of Akin et al. (2023) [27] has shown, most tourists visiting Turkey are wary of offal dishes served in ethnic restaurants [10,27]. In India, where there is a centuries-old tradition of vegetarianism, a lack of acceptance of offal consumption is observed [51]. Other reasons for low offal consumption have been identified in the U.S. [15]. At the root is consumer caution toward unfamiliar foods and reluctance due to revulsion [107,108]. Consumers in the U.S. unreasonably too often consider offal unsuitable for human consumption [109,110], despite the U.S. Department of Agriculture’s designation of offal as a safe, profitable, and valuable food [111]. Nevertheless, the number of pioneering sustainability-minded restaurateurs innovating with offal continues to grow [16]. Offal is used in cuisines all over the world [112], although it is clear that acceptance of the dish usually depends on the cultural and religious context [111].

Offal allows for a wide range of preparation methods leveraging its unique texture, composition, functional, and sensory properties [113]. It can be cooked whole, sliced, minced, or stuffed [19,36,51,103,113,114,115], and due to its distinctive sensory characteristics—such as taste, smell, and appearance—it is commonly used in the food industry to produce various meat products like pâté, liver sausage, black pudding, and brawn [19,20,22,37]. Offal also serves as an economical raw material [19,20,32]. At the same time, in many regions of the world, offal is the main ingredient in traditional products [25,32,116].

## 6. Nutritional Value of Edible Offal

Offal has historically been overlooked in dietary guidelines and recommendations [117], leaving consumers today with insufficient information about its nutritional value. Yet, a key attribute of offal is its often superior nutritional value compared to conventional meat cuts [20,22,33,118]. The chemical composition largely depends on the type of organ. It is recognized that foods of animal origin have a higher bioavailability of many nutrients [3,29,30]. Specifically, offal is rich in nutrient complexes such as folic acid, choline, and vitamin B12, boasting levels of bioavailability hard to match with other food sources [45].

Numerous studies [6,7,8,9,20,21,23,44,45,51,52,58,61,68,69,78,117,119] have shown that offal contains a significant amount of high-quality protein, minerals (including iron, phosphorus, copper, magnesium, iodine, calcium, potassium, sodium, selenium, zinc, and manganese), vitamins (such as B1, B2, B6, folic acid, B12, A, D, E, and K), and essential fatty acids, especially *n*-3 eicosapentaenoic acid (EPA) and docosahexaenoic acid (DHA), and have a favorable polyunsaturated fatty acid (PUFA) to saturated fatty acid (SFA) ratio. The chemical composition primarily determines the favorable taste and dietary qualities. Offal, except liver, contains few carbohydrates [56,69].

The liver stands out for its nutrient density [3,9,14,24,120,121]. Consuming 100 g of liver daily can fulfill up to 50% of the recommended intake for iron, zinc, selenium, B vitamins, and 100% of vitamin A [30]. Pork liver is particularly rich in nucleic acids, and the high purine content shapes the taste of liver [122]. It is generally accepted that foods high in purines are umami foods [24,123].

The kidney offers a valuable source of protein in a concentrated form, complete with all essential amino acids. It is also rich in vitamin B12, selenium, iron, copper, phosphorus, and zinc and contains a significant amount of saturated fatty acids, as well as *n*-3. Culinary experts believe that beef kidney has the mildest taste [45]. Spleen and lungs are considered to have lower consumer and commercial value due to their specific histological structures [59].

Most scientific publications focus on the chemical composition of what is known as red offal (liver, heart, kidneys, and tongue), whereas information on white offal (lungs, brain, stomach, and intestines) is less commonly found. There is even less information available on bird offal. The composition of offal is predominantly water, followed by protein and fat. Some studies have also examined the content of cholesterol and collagen (Table 2). Generally, water content is crucial in determining the quality and durability of these raw materials [124], as well as the proportion of other chemical components present in the offal.

### 6.1. Proteins and Amino Acids

The protein content in offal is high (Table 2), with a broad range from about 7% (in intestines) to approximately 30% (in wild boar liver). Red offal shows less variability in protein content than white offal. Notably, organic animals offer a rich source of proteins, comparable to those found in muscle tissue [9,14]. The liver typically has the highest protein content, while the intestines have the lowest. The protein content is influenced by the age of the animal—beef tongues contain more protein than lamb and calf tongues. Also, breed shapes the protein content of the kidneys, brain, heart, and spleen [22,32,59].

The housing system of pigs affects the protein content of the lungs and liver. The organs of pigs kept on a slatted floor have a higher proportion of protein compared to pigs kept on deep litter [69]. The heart, lungs, and liver of pigs contain less protein compared to the corresponding offal of wild boars [58,78]. In contrast, the season of the year does not affect the chemical composition and protein content of offal [74].

The protein content of the livers and hearts of chicken broilers from organic production systems is higher than that in the livers of birds from conventional production systems. The reason for the higher protein content in hearts is believed to be due to the higher motor activity of organically reared birds [35]. The ostrich liver contains significantly less protein than the heart and stomach [36].

Collagen, found in the form of connective tissue, is one of the proteins that shape the tenderness, digestibility, and nutritional value of raw materials [125]. However, the presence of this tissue in the liver (there is up to 3.3% of it in pork) limits its attractiveness in dishes [24]. Age, species, and breed affect the amount of collagen in offal [22,58]. Compared to pigs, wild boar hearts contain less, while livers contain more collagen. At the same time, wild boar livers contain twice as much collagen as kidneys and hearts, and three times less than tongue and lungs.

Essential amino acids, which have important physiological functions, must be supplied in the diet because they are not produced by the human body [126]. The high biological value of offal is due to a similar set of essential amino acids to muscle proteins. Moreover, they are not lost during thermal processing, since offal does not contain reducing sugars, which, along with amino acids, are the substrate of the Maillard reaction [127]. The content of essential amino acids in offal varies greatly (Table 3). Differences in the content and type of amino acids are due to the varying protein composition of offal, such as collagen content [20,66]. The highest levels of essential amino acids, such as threonine, valine, isoleucine, leucine, phenylalanine, lysine, and histidine were found in pork [20] and sheep [66] liver, making up nearly 50% of their total amino acid content [24].

However, the variation in amino acid content is not solely dependent on the species of the animal. Hoffman et al. (2013) [32] observed significant differences in the amino acid levels in the kidneys, heart, brain, and lungs among sheep of different breeds, though no such differences were noted in the amino acid content of the stomach and tongue.

### 6.2. Fat and Fatty Acid Profile

As a vital component of our diet, fat not only provides energy but also aids in the absorption of vitamins A, D, E, and K. Nonetheless, excessive daily consumption of fat is linked to a heightened risk of endocrine diseases, cardiovascular diseases, and obesity [128]. It is recommended that fats should constitute 20–35% of an adult’s energy needs, with less than 10% being SFAs (saturated fatty acids), 15–20% MUFAs (monounsaturated fatty acids), and 6–11% PUFAs (polyunsaturated fatty acids) [129]. Essential fatty acids like PUFAs *n*-6 and *n*-3, which are not synthesized by the human body, must be obtained through diet [20,116]. These fats are crucial for physiological functions, cell membrane operations, immune system performance, and the regulation of blood clotting. Offal, comprising various internal organs and entrails of animals, features a specific composition of fatty acids influenced by factors such as type, genetics, environment, the age of the animal, breed, diet, and overall fat content [116,117]. The ratio of SFAs and MUFAs escalates more rapidly than that of PUFAs as body fat increases, leading to a proportional decrease in PUFAs [37].

The proportion of fat in offal reported in publications by authors (Table 4) may be overestimated [117] and is generally at a level similar to or even lower than that in meat. The interaction of factors such as age, weight, sex, genotype, castration, and nutrition affect fat deposition and fatty acid profile in offal [116].

The liver, heart, kidneys, and lungs show particularly low fat content [22,61,117,130,131]. High fat content is characteristic of the tongues of sheep, lambs, and pigs [8,9,20,24,32,33,58,59,66,69]. Also, breed causes significant differences in the fat content of sheep [32] and pig [22] offal. This is especially true for the fat content of the tongue, heart, liver, and kidneys. Also, gender affects the fat content of pig liver. The liver of males contains more fat than that of females [58]. But the livers and hearts of male emus contain less fat than the livers of females [37].

The offal of wild animals and birds from open housing systems contains less fat than that of livestock. The low fat content is because the animals and birds can move freely over a large area, which reduces fat deposition in their internal organs [37]. Likewise, pigs kept on a grate floor have less fatty offal (heart and liver) compared to pigs kept in conditions that favor extended resting time [69]. Abdullah and Buchtova (2016) [35] noted that the fat content of the livers of organic broilers is significantly higher compared to that of conventional broilers.

Pork liver is exceptionally rich in phospholipids, second only to egg yolk and beef brain, with phosphatidylcholine constituting over half of these phospholipids [24,132]. Beef offal, including the kidney, lung, and heart, can notably enhance the absorption of the less bioavailable nonheme iron from vegetables, fortifiers, and supplements by about 200% due to the phospholipids or protein fraction naturally present in offal [68].

The indexes atherogenic (AI), thrombogenic (TI), and hypocholesterolemic/hypercholesterolemic (h/H) ratio are calculated to determine the nutritional quality fats of meat and other edible parts of animals. AI and TI express the impact of fatty acids on human health especially cardiovascular. AI indicates the risk of fat buildup in the artery walls, which can cause atherosclerosis, while TI indicates the possibility of blood clot formation [133]. The higher the values of these coefficients, the greater the risk of developing cardiovascular diseases [134]. The h/H ratio is associated with the functional activity of fatty acids in lipoprotein metabolism to transport cholesterol in plasma and with the risk of cardiovascular disease. Higher values of this indicator are more desirable [135].

Cholesterol, vital for the normal functioning of the body, is synthesized from saturated fats by the liver and other organs. While cholesterol is necessary for producing steroid hormones (such as cortisol, testosterone, and estrogen), fat-soluble vitamins (including vitamins A, D, E, and K), and bile acids, there can be excessive dietary intake of cholesterol, particularly from sources rich in saturated fats. Other factors, such as nutrition and the age of the animal, heavily influence the cholesterol content of offal. However, high cholesterol intake is also associated with an increased risk of cardiovascular diseases, including ischemic heart disease, hypertension, and diabetes. Therefore, moderate consumption of offal is advisable due to its cholesterol content [3,32,37,136,137].

#### 6.2.1. Saturated Fatty Acids (SFAs)

Dietary SFAs are known to elevate plasma lipid levels, increasing total cholesterol and low-density lipoprotein (LDL) concentrations, which heightens the risk of metabolic (insulin resistance, obesity, type 2 diabetes) and cardiovascular diseases [138,139].

The SFA content varies with the age of the animal; for instance, calf offal contains more SFAs like lauric (C12:0), myristic (C14:0), and palmitic acid (C16:0) but less stearic (C18:0) and arachidic acid C20:0 compared to adult cow offal. The highest C18:0 content was found in the adult liver [13,14]. The highest percentage of SFA is found in the liver, kidney, and tongue of calves and the liver of cows. The age of cattle determines the SFA content of hearts, kidneys, and tongues [14].

In lamb and sheep offal, SFAs constitute 45–70% of the fatty acids, unaffected by the sheep’s age [32,116,117]. Interestingly, the content of pentadecanoic acid (C15:0), indicative of long-term milk consumption, is significantly higher in lamb offal than in sheep offal. In contrast, the proportion of SFAs depends on the type of organ, ranging from 47 to 48% in lamb and mutton tongue to 66–67% in hearts and stomachs. Interestingly, lamb and mutton tongues have the lowest percentage of SFAs and the highest percentage of MUFAs [117]. SFA content does not depend on the breed of sheep [32].

Sheep offal, especially ram’s liver and tongue (1791 mg/100 g), is particularly rich in cholesterol, and therefore, its consumption should be limited in the human diet [32]. The lowest amount of cholesterol is found in lamb lungs (12 mg/100 g) [117].

The fatty acid profile in pork and wild boar offal depends primarily on the type of organ. In most cases, SFA content of more than 40% is found, and these SFAs are mainly C16:0 and C18:0. The lowest proportion of SFAs is found in the heart [20,116]. The highest content of C18:0 is found in the liver. Of the remaining SFAs, a higher than 1% content of C14:0 was found in all offals except the liver [58].

In the case of red deer, most SFAs were found in liver fat [87]. As in the offal of the other animals discussed above, C16:0 and C18:0 acids dominate among the SFAs, with the highest proportion of C18:0 found in the liver. The skeletal muscles of deer contain more C16:0, while the offal (heart, liver, and kidney) contains more C18:0. Similar ranges of C18:0 percentages were found for bovine offal by other authors, except for kidneys [14]. The kidneys of domestic cattle, unlike those of red deer, have a lower proportion of C18:0 than C16:0. Red deer offal also has a higher proportion of other SFAs, especially behenic acid (C22:0) [87].

Bird offal is similarly dominated by SFAs [37,140], with the liver having the highest content, followed by the stomach and heart [37,115,141,142]. The fatty acid profile in birds is led by C16:0 and C18:0, with the largest amounts of C16:0 found in emu livers and hearts [37] and poultry stomachs [34]. The liver contains the most C18:0, with slightly lesser amounts in bird stomachs and hearts [34,37]. The gender of emus does not influence the cholesterol levels in their offal [37]; the highest cholesterol level is present in the liver, followed by the stomach and heart. Compared in concentration, emu offal is most akin to broiler livers but has higher cholesterol than ostrich and turkey livers (329.33 mg/100 g) [143].

Nutritionally, it is recommended that pork offal be consumed in limited quantities and as part of a diverse and balanced diet [116].

#### 6.2.2. Unsaturated Fatty Acids (PUFAs and MUFAs)

The consumption of unsaturated fatty acids is believed to offer health benefits [32], and although SFAs and PUFAs are ingested together, the ideal ratio should not exceed 0.4 [144]. Offal typically contains more MUFAs than PUFAs [117]. Offal is a source of essential fatty acids like linoleic acid (C18:2*n*-6) and alpha-linolenic acid (C18:3*n*-3), in addition to PUFAs such as arachidonic acid (C20:4*n*-6) and eicosapentaenoic acid (C20:5n3), which are crucial in preventing chronic diseases, including ischemic heart disease and certain cancers. However, it is important to note that the high unsaturated fatty acid (UFA) content in offal makes it susceptible to oxidation during heat treatment and storage [58].

The fatty acid profile in calf offal is significantly influenced by the milk’s composition, particularly from cows that graze on pastures, due to its rich PUFA content (CLAs—conjugated linoleic acids, C18:3, C20:2, and C20:3) [14]. This composition endows calf offal with various potential health benefits, including the ability to lower total blood cholesterol levels and exhibit anticancer, antidiabetic, immunomodulatory, and antiobesity effects [145,146]. CLAs (cis-9, trans-11 18:2; 18:2c9t11) dominate the naturally occurring isomers in offal from grass-fed ruminants. They are formed by microbial fermentation of PUFAs and the isomerization of linoleic acid, with cow offal displaying significantly higher CLA levels compared to calf offal. Bovine tongues are noted for their higher MUFA content, primarily due to elevated levels of C16:1 and C18:1, whereas the lowest C18:1 concentration is observed in the hearts and livers of cows. Among offal types, cattle hearts are particularly high in C15:1, while beef offal showcases the highest levels of C18:2 and C20:4, with hearts rich in C18:2 and kidneys in C20:4 [14].

In sheep, the proportions of SFAs, PUFAs, and MUFAs are largely age-dependent. MUFAs in sheep offal make up 25 to 50% of total fatty acids, generally surpassing those in lamb offal [117]. Notably, sheep hearts have fewer MUFAs compared to tongues, and sheep breed does not influence MUFA and PUFA levels. Oleic acid (C18:1n9c), which is prevalent in all offal types, with tongues having the highest concentration, is known to lower blood pressure in hypertensive patients and may hinder the pathogenesis of adrenoleukodystrophy (ALD) [147]. High levels of nervonic acid (C24:1n9) and DHA (C22:6n3) in sheep brains offer therapeutic benefits in treating demyelinating diseases such as multiple sclerosis and ALD [32]. The PUFA content varies by offal type, with linoleic acid (C16:2n6c) being the most abundant PUFA [117] and the lowest PUFA content being found in the tongue [32]. The liver and kidney predominantly contain C20:4*n*-6, while the liver also has high levels of C22:5*n*-3, C18:2*n*-6, and DHA (C22:6*n*-3), and the kidney is rich in C18:2*n*-6, C22:5*n*-3, and C20:5*n*-3. Lamb offal exhibits a higher CLA content than sheep offal [117]. The presence of oleic acid enhances the nutritional value of offal [148]. Sheep offal showed a higher content of essential fatty acids compared to lamb offal [117]. The PUFA/SFA ratio shows considerable variability in sheep offal, ranging from about 0.03 (hearts and tongues, stomachs, and intestines) to 0.49 (brains) [32,117]. Sheep brains contain twice as many *n*-3 fatty acids as *n*-6, mainly C22:6n3c, C22:6n3c, C18:3n3c, and C20:5n3c. The *n*-3 fatty acids are associated with a reduced incidence of cardiovascular disease. The *n*-6 fatty acids (C20:4n6, C18:3n6, and C18:2n6c), which are blamed for chronic inflammation in heart disease, diabetes, and cancer, are found in offal in fairly small amounts [32,117]. Sheep offal has a higher content of cholesterol-lowering rather than cholesterol-raising fatty acids, although the proportion of these fatty acids depends on the type of offal and the age of the animal [117].

The fatty acid composition of pork offal depends on its type (Table 4). The heart, liver, and stomach are the organs with the highest total content of unsaturated fatty acids. Oleic (C18:1c9), stearic (C18:0), palmitic (C16:0), vaccenic (C18:1*n*-7), linoleic (C18:2*n*-6), arachidonic (C20:4*n*-6), and elaidic (C18:1n9t) acids have the highest proportion. The largest proportion of MUFAs is found in the tongue. The tongue, brain, and pancreas contain the most C18:1c9. The heart, kidney, and liver are highlighted for their higher values of PUFAs and *n*-6 fatty acids. Except for the brain, the PUFA *n*-3 content in pork offal is relatively low, albeit higher than in muscle tissue, indicating a distinct fatty acid profile within these organs [149,150]. Notably, significant differences exist in the PUFA/SFA and *n*-6/*n*-3 ratios across different types of pork offal. The heart and kidney showcase the most favorable PUFA/SFA ratios [20,151]. Meanwhile, the *n*-6/*n*-3 ratio spans a wide range, from 8.57 in the liver to 90.61 in the spleen [152,153,154], suggesting that some offal types offer a more favorable fatty acid profile than meat [20].

Wild boar offal is distinguished by a more favorable fatty acid profile when compared to pork offal. It features higher levels of UFAs and boasts a favorable PUFA/SFA ratio (>0.4%). Additionally, wild boar offal contains significantly higher levels of C18:1 isomers. The highest PUFA levels were observed in pig liver and boar heart, with the content of linoleic (C18:2n6c) and linolenic (C18:2n6t) acid in the boar heart and kidney nearly double that found in pigs. Conversely, pig offal has a greater amount of arachidonic acid than wild boar offal. The content of neutral and hypocholesterolemic fatty acids (UFAs + C18:0), which are thought to reduce total cholesterol levels, constituted 61–79% of the total DFAs, highlighting their potential health benefits [58].

In red deer offal, like wild boar offal, the primary MUFAs include oleic acid C18:1*n*-9), vaccenic acid (C18:1*n*-7), and oleopalmitic acid isomers (C16:1*n*-9 and C16:1*n*-7) [87]. The PUFA/SFA ratio in red deer offal is notably higher than the recommended minimum, presenting a more favorable profile than that found in domestic ruminants.

The fatty acid composition within bird offal varies significantly depending on the organ and bird species, particularly in the proportions of MUFAs and PUFAs, which display considerable variability [34,37,115,141,142,155,156]. In emu offal, the MUFA fraction is predominantly composed of oleic (C18:1n9c), elaidic (C18:1n9t), and palmitic (C16:1) acids, while the PUFA fraction chiefly consists of arachidonic (C20:4n6) and linolenic (C18:2n6c) acids. Notably, emu offal contains a higher amount of the beneficial arachidonic acid compared to other bird species. Oleic acid is prevalent in the livers of various poultry, with its proportion in the lipid fraction ranging widely, from a few percent to over 40% [34,115,140,141,142,156,157]. The most significant concentrations of this acid have been identified in the livers [115,140,141,142], stomachs [37], and hearts of birds [34].

### 6.3. Bioactive Substances and Vitamins

Offal is rich in a variety of biologically active substances, including L-carnitine [158,159], creatine, carnosine, taurine, coenzyme Q10 [160,161], anserine [162], glutathione [163], conjugated linoleic acid diene [164], and bioactive peptides [165]. These substances are typically found in higher concentrations in offal than in muscle meat [15,166]. The array of biologically active compounds present in offal offers multiple health benefits, such as antioxidant, anticancer, antihypertensive, anti-inflammatory, anticoagulant, cytomodulatory, and immunomodulatory effects, as well as boosting metabolism, enhancing digestion, and contributing to the reduction in blood glucose levels [4,18,32,167,168].

Coenzyme Q10, also known as ubiquinone, is a vital bioactive compound found across a wide range of animal organs [169], with particularly high concentrations in the heart, where levels can range from 113 to 192 µg/g [170,171]. The main function of ubiquinone is to transport electrons in the respiratory chain within the mitochondrion [14]. It also exhibits antioxidant activity, protecting lipids, proteins, and the LDL fraction of cholesterol from oxidation, as well as being synergistic with other antioxidants [169]. Ubiquinone reduces proinflammatory cytokines and blood viscosity, which is helpful for patients with heart failure and coronary artery disease and shows immune system-boosting properties [169,172,173].

Peptides, which are specific fragments of proteins, play a critical role in regulating cellular and intercellular physiological responses. While these peptides are often inactive within the structure of native proteins, they can be liberated during digestion in the gastrointestinal tract or through food processing methods such as enzymatic hydrolysis, bacterial fermentation, or in vivo digestion [174,175,176]. The biological activity of these bioactive peptides is influenced by their amino acid composition and sequence [53,177]. Offal is a rich source of such peptides, boasting properties that are antihypertensive, antimicrobial, antioxidant, immunomodulatory, anticoagulant, and even opioid in nature. These bioactive peptides have the potential to impact a range of bodily systems, including cardiovascular, immune, nervous, and digestive systems [18], and may play a key role in the prevention and treatment of conditions such as cancer, metabolic diseases, and mental health disorders [4].

The presence and concentration of these bioactive substances in meat and offal are influenced by various factors, including the species and breed of the animals, their feeding system, and the subsequent processing of the raw material [168,178]. Unfortunately, the available literature lacks information on the content of these substances in offal.

Vitamins represent a diverse group of exogenous organic compounds essential for the proper functioning of living organisms. They are crucial for health, albeit not to serve as direct sources of energy or cellular building materials. Offal, known for its myriad functions within the body, emerges as an exceptionally rich source of various vitamins, often surpassing skeletal muscle in nutritional value [20,179]. However, literature on this subject remains somewhat limited. The obtained numerical data are presented in Table 5.

Incorporating red lamb offal into the diet can significantly enhance the intake of vitamin A (retinol), vitamin B12, folic acid, and vitamin C, more so than other parts of the carcass. Relative to its weight, offal contains higher amounts of thiamine, riboflavin, niacin derivatives, and vitamin B6. The liver, notably, stands out for its vitamin richness, providing 99% of the total vitamin A, 71% of folic acid equivalents, 63% of vitamin B12, 41% of vitamin C, and 34% of riboflavin, relative to the entire carcass weight. Kidneys contribute 7% of the total vitamin B12 with less than 1% of the carcass weight, while lungs offer 31% of the total vitamin C, accounting for only 2% of the carcass weight [3].

The content of B vitamins in offal is on par with that in processed cereals and soybeans, which are celebrated as top sources of these nutrients [20,198]. Vitamin B1, found in the liver, kidneys, heart, and brain, enhances the efficiency of inhibitory transmitters in the nociceptive system and boosts the analgesic effects of non-opioid pain relievers [199].

Vitamin B2, abundant in the liver, kidneys, and heart, activates enzymes involved in metabolizing antidepressants. Niacin, predominantly located in the liver, kidneys, heart, and brain, serves as a coenzyme for various oxidation–reduction reactions, playing a key role in cellular protection [199]. Vitamin B6 acts as a coenzyme in reactions related to the metabolism of Chinese hamster ovary cells, lipids, neurotransmitters, and amino acids [199]. Pantothenic acid, part of coenzyme A, is crucial for metabolism and acts as a carrier protein in fatty acid synthesis [200]. Biotin, mainly found in the liver and kidneys, participates in carboxylation and transcarboxylation reactions. The liver is also a significant source of vitamin E, which prevents lipid peroxidation and supports anti-aging efforts, and it synergizes with anticoagulant medications [18].

Folate encompasses a group of vital exogenous compounds that play crucial roles in numerous biological processes, including the synthesis of nucleic and amino acids. It is indispensable for the healthy operation of the nervous, hematopoietic, and circulatory systems. Folate deficiency is linked to several health issues, such as megaloblastic anemia, hyperhomocysteinemia, and potentially cancer [201]. Especially in the prenatal period, it can cause miscarriages, and the body of the developing fetus can develop neural tube defects and handicap the child [202]. Despite the importance of folic acid for the proper functioning of the body, its deficiency in the diet is a common phenomenon worldwide [203]. The European Food Safety Authority (EFSA) [204] recommends a daily folate intake of 330 µg. The liver, being the primary folate storage organ in the body, along with processed liver products like pâté, presents a highly bioavailable dietary source of folate. The main organ that stores folate in the body is the liver. Therefore liver and processed foods (e.g., pâté) are a good dietary source of folic acid [205] with very high bioavailability [206]. Nevertheless, research on folate content in offal is limited and often lacks detail regarding the types of folates, their bioavailability, and the influence of factors like breed, diet, age, or slaughtering methods on folate levels. Animal livers, especially poultry livers with up to 1.3 mg of folate/100 g of fresh liver, alongside their high bioavailability and rich content of other nutritionally valuable substances, underscore the necessity for more comprehensive research in this domain [180].

Vitamin B12, exclusively found in animal-derived products, is crucial for erythrocyte production, thymine synthesis, DNA formation, and proper cell division. It facilitates the intestinal absorption of intrinsic vitamin factors, enhancing the oral delivery of peptides and proteins as vitamin B12 conjugates [199,200]. Offals are natural, very good sources of vitamin B12, especially offal from ruminants due to the biosynthesis of cobalamin by the bacteria and archaebacteria inhabiting their digestive tract [207]. Vitamin B12 is absorbed in the gastrointestinal system and transported via the bloodstream to organs like the liver and kidneys [188]. The active forms of vitamin B12, adenosylcobalamin (coenzyme B12), and methylcobalamin are essential for various bodily functions [208]. EFSA’s (2015) [209] daily recommendation for vitamin B12 intake stands at 4.0 µg. The concentration of vitamin B12 in offal depends on the composition and type of feed. The content of vitamin B12 in the livers of various animals ranges from 59.0 to 110.0 μg/100 g for beef and 60.0 μg/100 g for veal. The amount of this vitamin is 23.0–28.0 μg/100 g in calf kidneys and 27.0–31.0 μg/100 g in beef kidneys.

Vitamin D, a fat-soluble compound within the secosterol group, is naturally present in a limited number of foods, primarily those of animal origin, in the form of vitamin D3 (cholecalciferol). It serves as a prohormone and is converted into its biologically active form, 1,25(OH)2D (1,25-dihydroxyvitamin D), through hydroxylation processes in the liver and kidneys. Circulating vitamin D primarily exists as 25(OH)D (25-hydroxycholecalciferol, calcidiol), a metabolite produced by the first hydroxylation of the vitamin D3 molecule at the 25th position in the liver. Calcidiol exhibits limited biological activity and is further hydroxylated in the kidneys to produce 1,25(OH)2D, the active vitamin D hormone (calcitriol). Consequently, the liver, particularly due to its vitamin D content, is considered a valuable dietary source, notably superior to lean meat [210,211,212]. EFSA in 2012 [213] recommended a daily intake of 100 µg/day of vitamin D for adults. The livers and kidneys of cattle and pigs are dietary-rich and highly bioavailable sources of vitamin D.

Vitamin A predominantly occurs in the liver as retinol and retinol esters, concentrated forms of this vitamin. Retinoid, an unsaturated hydrocarbon featuring four isoprenoid units, exhibits pharmacological effects crucial for cell protein metabolism, epithelial integrity, and vision [199].

Furthermore, the liver is enriched with taurine, which plays a role in regulating immune dysfunction, and choline, a precursor to phospholipids and neurotransmitters that safeguard the liver against oxidative stress. A diet sufficient in vitamin E and *n*-3 fatty acids has been shown to alleviate pain associated with menstrual cramps [214] and cyclic breast pain in women [215].

### 6.4. Minerals

A crucial determinant of human well-being is a balanced diet, providing appropriate amounts of essential elements—both macronutrients and micronutrients. Micronutrients, chemical elements required in amounts less than 100 mg/day, are vital for the body’s proper functioning. Conversely, the need for macronutrients, which are not synthesized by the human body, is higher, necessitating dietary intake [20,51]. The prevalence of diet-related deficiencies and diseases remains a significant issue, particularly in famine-stricken countries. Daily nutritional requirements vary based on age, gender, and physiological status. Both micronutrients and macronutrients perform diverse nutritional (contributing to the building blocks of bones and teeth) and physiological (acting as components of electrolytes and enzymes, serving as catalysts) roles within the body. They also affect the taste, color, and texture of food. Generally, offal contains a higher mineral content compared to muscle tissue.

Foods of plant origin generally contain fewer of these compounds or have them in forms that are less readily absorbed by the human body. The content of macronutrients and micronutrients in offal varies widely, influenced by factors such as species, breed, age, sex, physiological state, mode of maintenance and nutrition [119,216,217], and the specific type of offal [33]. The natural variation in nutrient content is unclear [217]. Overall, offal is a valuable source of minerals, especially zinc, iron, copper, and selenium (Table 6). Zinc is essential for numerous fundamental biochemical and physiological processes at the cellular level in humans [218,219,220,221,222]. Its deficiency can lead to anemia, motor dysfunction, and appetite disorders, among other issues, while an excess can also be detrimental to health [45,223]. The daily requirement ranges from 8 to 18 mg. Iron is crucial for hemoproteins and is involved in proteins related to oxidative phosphorylation and iron accumulation [224,225]. It is integral to myoglobin molecules, which distribute oxygen to tissues. Both deficiency and excess of iron can cause significant disruptions in bodily functions [24]. The daily requirement for iron is also 8–18 mg. Copper serves as a vital catalytic cofactor for around 300 enzymes involved in redox reactions crucial to basic biological functions [51,226], including germ cell production and oxidoreductive reactions. Both excessive intake and deficiencies of copper (and zinc) can affect iron retention [24,226], leading to stunted growth, bone demineralization, and heart and gastrointestinal diseases [227,228]. The daily requirement for copper ranges from 340 to 890 µg. Selenium is vital for the thyroid gland, the immune system, and the activity of glutathione and peroxidase group enzymes [229]. A selenium-deficient diet can lead to type 2 diabetes and cardiovascular and lung diseases [230], with a daily requirement of 15–55 µg.

Red offal typically contains higher levels of macronutrients and micronutrients compared to white offal [61]. However, certain physiological conditions cause minerals to accumulate more in specific pieces of offal, such as the liver and kidney [24], making them, along with the spleen, the offal type with the highest ash content [20,24,33,58,130,237]. Hearts and stomachs contain slightly less ash [37,238,239].

The liver is notably rich in micronutrients (Fe > Zn > Cu > Mn) and macronutrients (K > Na > Mg > Ca). Kidneys and lungs have the highest concentrations of Na and Ca [51,69,234,235,240]. Sodium plays a role in regulating water and electrolyte metabolism and assists in the transport of amino acids and carbohydrates to tissues. It works in synergy with potassium to form a gradient on both sides of the cell membrane, facilitating nerve impulse transmission and the contraction and relaxation of muscle cells [79]. The daily sodium requirement is up to 5 g. Potassium is crucial for blood pressure regulation, kidney function, water–electrolyte balance, nerve impulse conduction, and metabolic processes. Potassium deficiency can lead to irregular heartbeat, edema, and hypertension, with a daily requirement ranging from 1.5 to 3.5 g. Calcium is essential for building and regulatory functions, including the formation and maintenance of bones and teeth, blood clotting, muscle contraction, nerve impulse transmission, and cell membrane permeability. It also activates enzymes and aids in vitamin B12 absorption. Both deficiency and excess of calcium can lead to health issues, with a daily requirement of 0.8–1.3 g.

Manganese is crucial for the proper functioning of the nervous system and acts as a component of enzymes that are involved in the digestion and absorption of carbohydrates, lipids, and proteins, as well as energy production. The body is protected from manganese overdose by a homeostatic mechanism [45]. The daily requirement is in the range of 1.8–2.3 mg. The highest amount of Mn, like Zn, is contained in the pancreas. The lowest mineral content is found in the lungs, stomach, and intestines [20,69,241]. In the kidneys, the concentration of manganese is much higher than that in the liver, and the concentration of zinc is lower [3,242,243]. The main source of Mn in the human diet is pork liver [235]. Among offal, the liver is the richest source of Ca [119,244]. However, the liver and spleen are undoubtedly the richest sources of nonheme iron [119], although 20–25% of iron is also in heme form [24]. This is important because the human body absorbs heme iron more easily than nonheme iron [240,245]. The liver’s iron content far exceeds that of zinc, which is high anyway. With some exceptions [235], this is consistent with what is most often reported in the literature [24,45,118,130]. Emu liver is indicated as the richest dietary source of iron [115]. The highest amount of selenium is found in the livers of slaughtered animals—up to 80 µg/100 g [236].

The recommended dietary intake of micronutrients varies with an individual’s age and gender. Assuming the average daily intake for Fe, Zn, Cu, and Mn per adult is 13.0, 9.0, 0.9, and 2.1 mg/day, respectively [221], consuming 100 g of liver can fulfill approximately 154%, 55%, 87%, and 14% of these micronutrient requirements. Notably, the iron in meat is heme iron, which the body absorbs much more efficiently than nonheme iron from plant sources [20,58,149,246]. Pork liver provides 15 times more iron than pork meat, and liver from wild boar offers twice as much iron as pork liver [79]. Boar kidneys also have slightly higher levels of Zn and Cu compared to pork kidneys [247]. The liver is an abundant source of copper [51,58,248]; however, copper’s bioavailability from the liver of monogastric mammals is relatively low [249], but it is considerably higher in the livers of birds and ruminants [250]. Zinc in the diet acts antagonistically in the absorption of copper by the body [235,249].

The ash content in offal varies significantly by type [20,51,235]. Gender appears to have a marginal influence, though it is not entirely negligible [58,119]. Potassium is the predominant macronutrient in offal [37,115,239], with the spleen and liver being particularly rich in it, whereas magnesium is more abundant in hearts [20,69,241]. Magnesium plays roles in the formation and maintenance of healthy bones and joints, protein synthesis, and energy production; has a calming effect; and is involved in nerve conduction. Symptoms of magnesium deficiency can include fatigue, headaches, concentration issues, muscle cramps, and brittle hair and nails, with a daily requirement ranging from 250 to 400 mg. The content and composition of ash in offal also depend on the animal’s housing system [69].

## 7. Summary

Edible offal, the nonmeat parts of animals, emerges as a significant source of nutrients and functional substances in human diets, though often underappreciated in nutritional discussions. This review reveals the latest research findings on offal’s nutritional value, the potential impact on human health, and its sustainability impact.

Offal stands out as a rich source of high-quality protein, along with essential vitamins and micronutrients such as B vitamins, vitamin A, and highly bioavailable forms of iron and zinc. The research summarized in this paper illustrates that offal’s nutrient content can rival or even surpass that of animal skeletal muscle in concentration. Incorporating offal into diets can foster nutritional diversity and balance, particularly in developing countries where nutrient deficiencies are common. The nutrient profile and size of offal are significantly influenced by factors like the animal’s species, breed, age, and farming conditions, with the type of organ being of utmost importance. However, it should be remembered that edible animal offal may contain xenobiotic compounds, such as heavy metals, or if the hygiene of its production is insufficient, it may contain parasites (which were not discussed in this review). Therefore, a risk assessment of existing and potential hazards should be conducted every time. Nevertheless, due to its potent nutrient concentrations, especially cholesterol, offal consumption should be judicious to avoid surpassing the dietary intake limit. The solution may be an effective combination of different organs leading to a personalized/balanced diet with health-promoting qualities.

The importance of offal in culinary traditions varies in different parts of the world. Educating and promoting knowledge of its nutritional values could boost consumption, enrich culinary traditions, and even mitigate global hunger to some extent. Additionally, integrating offal into diets can contribute to reducing food waste through the efficient use of the whole animal. The use of offal in the diet reduces the amount of organic waste generated by the meat industry and reduces the need for disposal, which contributes to reducing the negative impact on the environment and increases economic efficiency. Promoting the consumption of offal can also have a positive impact on the environment by reducing greenhouse gas emissions associated with meat production. Animal offal has a complex impact on the sphere of human life, but most importantly, as it has been shown, it is a valuable alternative to meat in the diet contributing to, among others, the diversification of sources of nutrients, as well as reducing pressure on natural ecosystems.

## Figures and Tables

**Table 2:** Proximate composition of edible offal (% by weight of edible offal).

Animals	Edible Offal	Moisture	Minerals	Protein	Lipids	Collagen	References
Cattle	Liver	70.0–74.7	1.30–1.71	19.50–21.12	2.54–4.05	nd	[9,14,61,68]
	Heart	76.3–79.3	1.00–1.56	17.25–19.9	1.05–3.95	nd	
	Kidney	74.4–79.04	1.20–1.81	15.5–18.1	1.51–6.09	nd	
	Tongue	71.5–77.0	0.92–1.19	17.3–19.05	3.22–10.3	nd	
	Lung	79.8	1.05	17.3–21.12	1.82	nd	
	Brain	76.29	1.10	10.86	10.3	nd	
Calves	Liver	70.59–70.73	1.35–1.52	19.36–22.11	3.14–4.75	nd	[9,14]
	Heart	77.0–78.52	1.10–1.23	17.15–18.74	1.51–3.95	nd	
	Kidney	79.0–79.03	1.10–1.35	15.7–17.53	1.89–3.12	nd	
	Tongue	74.5–75.14	1.00–1.02	17.15–17.22	5.32–5.45	nd	
	Brain	80	1.3	10.15	5.15	nd	
Sheep	Liver	66.7–69.9	1.6–1.9	18.8–20.9	4.3–11.8	nd	[8,32,66]
	Heart	66.8–70.3	0.9–1.1	13.5–17.2	11.7–16.4	nd	
	Kidney	76.7–80.5	1.03–1.7	14.9–16.2	1.1–5.2	nd	
	Tongue	64.1–66.2	0.61–0.9	12.9–15.2	11.8–21.7	nd	
	Lung	77.9–79.3	1.08–1.1	15.1–17.6	1.79–4.6	nd	
	Spleen	77.2–75.3	1.34–1.7	16.1–20.4	2.86–4.3	nd	
	Stomach	70.9–82.7	0.39–0.9	10.3–15.5	1.70–18.5	nd	
	Intestines	64.5	0.28	6.96	28.6	nd	
	Brain	77.8–78.0	2.0	8.70–10.10	10.10–11.9	nd	
Lamb	Liver	61.2–71	1.4	18.5–20.4	5.2–8.9	nd	[8,9]
	Heart	65.1–76	0.87–1.1	16.27–17.2	5.68–11.8	nd	
	Kidney	63.8–79	1.1–1.3	15.2–15.5	2.85–3.2	nd	
	Tongue	55.5–63.7	0.83–0.9	16.0–15.5	17.15–17.7	nd	
	Lung	74.1	1.05	17.3	2.5	nd	
	Spleen	67.1–79.5	1.18–1.2	7.91–10.5	2.00–8.58	nd	
	Stomach	49.6	0.43	10.0	15.7	nd	
	Intestines	55.2	0.46	7.01	26.5	nd	
Pig	Liver	63.3–74.5	1.3–1.67	16.60–26.31	1.76–8.11	0.7–1.77	[20,22,24,33,58,59,66,69]
	Heart	69.93–75.94	0.81–1.02	15.7–17.62	2.69–8.40	2.13–2.22	
	Kidney	73.8–80.15	1.08–1.2	13.47–18.87	3.12–4.93	1.46–1.86	
	Tongue	63.55–69.25	0.79–1.01	14.79–17.06	11.18–20.49	2.73–2.81	
	Lung	74.9–81.67	0.76–1.12	12.97–21.16	1.79–6.11	2.81–3.3	
	Spleen	78.58–79.37	1.12–1.36	17.15–17.79	0.97–1.80	nd	
	Stomach	77.63	0.31	17.07	4.05	nd	
	Small intestine	82.48	0.3	11.99	1.16	nd	
	Large intestine	69.73	0.15	8.45	19.54	nd	
	Pancreas	72.2	1.26	20.98	7.18	nd	
	Brain	76.46	1.43	10.71	8.71	nd	
Wild animals: wild boar, fallow deer	Liver	58.1–79.23	1.31–1.33	17.79–29.86	1.38–7.82	1.47	[58,74,78]
	Heart	72.06–72.73	1.11–1.13	19.06–20.8	1.47–3.05	1.69	
	Kidney	70.33–80.95	1.26–1.29	15.97–20.19	1.07–4.84	1.64	
	Tongue	67.46–68.42	1.01–1.04	15.25–16.66	12.56–14.77	2.71	
	Lungs	66.32		25.08	6.65	2.33	
Birds: ostrich, emu, turkey, chicken	Liver	64.2–75.44	1.10–1.74	16.6–19.95	1.7–14.3	nd	[35,36,37]
	Heart	74.56–79.61	0.98–1.2	13.77–18.5	0.7–6.97	nd	
	Stomach	77.7–80.23	0.88–1.1	17.1–19.0	0.74–3.2	nd	

nd—no data.

**Table 3:** Content of exogenous amino acids in offal.

Animal	Edible Offal	Methionine	Threonine	Valine	Isoleucine	Leucine	Phenylalanine	Lysine	Histidine	References
Pig *	Liver	0.36–0.91	0.45–0.70	1.12–2.01	0.91–1.02	1.05–1.61	0.91–1.29	1.06–1.33	0.69–1.10	[20,66]
	Heart	0.21–0.46	0.39–0.55	0.69–1.52	0.53–0.76	0.10–0.71	0.55–0.94	0.90–1.05	0.56–0.72	
	Kidney	0.48	0.48	0.66	0.49	0.92	0.52	0.64	0.49	
	Lung	0.20–0.40	0.22–0.46	0.73–1.31	0.42–0.61	0.72–0.89	0.50–1.61	0.72–1.17	0.54–0.79	
	Spleen	0.23	0.32	1.54	0.75	0.80	1.07	1.05	0.79	
	Stomach	0.21	0.33	1.42	0.71	0.80	1.63	1.06	0.66	
Sheep *	Liver	0.77	0.59	0.94	0.64	1.21	0.71	0.77	0.60	[66]
	Heart	0.70	0.62	0.79	0.34	1.19	0.62	1.13	0.65	
	Kidney	0.44	0.46	0.66	0.51	1.07	0.54	0.68	0.46	
	Lung	0.42	0.43	0.65	0.39	0.88	0.49	0.60	0.46	
Sheep **	Liver	1.3–1.4	3.0–3.3	3.4–3.7	2.8–3.2	7.4–8.2	3.7–4.1	5.3–5.6	2.1–2.2	[32]
	Heart	1.1–1.2	2.2–2.5	2.1–2.4	1.8–2.1	5.3–4.7	1.9–2.1	3.5–4.0	1.3–1.4	
	Kidney	1.4–1.5	2.1–3.3	3.3–3.6	2.6–2.9	6.9–7.7	3.1–3.5	4.3–5.0	1.6–2.0	
	Tongue	1.1–1.2	2.3	2.3–2.5	1.9	5.2	2.1–2.3	4.9–5.1	1.2	
	Lung	1.2–1.3	3.0–3.5	3.6–3.9	2.0–2.2	7.4–8.2	3.1–3.4	5.6–5.9	2.1–2.5	
	Spleen	1.3	2.8	3.4–3.5	2.2–2.5	7.3	3.3–3.4	5.3–5.4	2.1–2.3	
	Stomach	1.2–1.3	2.6–2.8	2.6–2.8	2.0–2.2	5.8	2.4	5.0–5.1	1.2–1.3	

* Content in % by weight of edible offal; ** content in g of 100 g dry, defatted matter.

**Table 4:** Composition of fatty acids and indicators of the nutritional quality of fats.

Animal	Edible Offal	SFA (%)	MUFA (%)	PUFA (%)	MUFA:SFA	PUFA: SFA	h/H *	*n*-6/*n*-3	AI	TI	Cholesterol (mg/100 g)	References
Cattle *	Liver	49.87	18.71	31.42	0.38	0.63	2.98	nd	nd	nd	335.7	[14,61]
	Heart	33.09	22.03	44.88	0.67	1.41	4.42	nd	nd	nd	170.1	
	Kidney	39.66	23.60	36.74	0.59	0.93	2.68	nd	nd	nd	502.3	
	Tongue	42.91	46.82	10.25	1.09	0.24	1.62	nd	nd	nd	728.6	
	Lung	nd	nd	nd	nd	nd	nd	nd	nd	nd	603.9	
Calves *	Liver	51.44	24.86	23.69	0.48	0.46	1.67	nd	nd	nd	nd	[14]
	Heart	37.57	29.83	32.59	0.80	0.90	2.83	nd	nd	nd	nd	
	Kidneys	49.97	30.11	19.92	0.60	0.44	1.59	nd	nd	nd	nd	
	Tongue	49.69	40.04	6.27	0.89	0.13	1.12	nd	nd	nd	nd	
Sheep *	Liver	47.2–51.0	24.1–33.0	18.9–24.0	nd	0.4–0.5	nd	6.0–7.3	nd	nd	168.2–205.5	[32]
	Heart	68.2–70.0	21.1–26.8	1.8–7.3	nd	0.0–0.1	nd	9.0–20.4	nd	nd	48.6–56.5	
	Kidney	45.4–46.5	30.2–31.8	20.2–21.2	nd	0.5	nd	10.2–11.4	nd	nd	155.6–228.2	
	Tongue	44.5–51.9	43.5–50.6	2.2–3.7	nd	0.0–0.1	nd	7.8–18.9	nd	nd	46.6–51.3	
	Lung	47.7–51.9	27.7–28.7	18.1–21.5	nd	0.4–0.5	nd	4.1	nd	nd	175.7–201.2	
	Spleen	52.3–53.9	28.2–30.6	14.6–14.9	nd	0.3	nd	7.4–7.5	nd	nd	177.4–188.2	
	Stomach	50.2–51.5	37.6–38.9	8.0–8.4	nd	0.2	nd	9.5–13.4	nd	nd	30.9–35.8	
Sheep **	Liver	2.68	1.18	0.27	nd	0.11	nd	12.50	0.82	2.85	26	[117]
	Heart	8.32	3.20	0.58	nd	0.08	nd	9.66	1.15	3.65	786	
	Tongue	9.68	9.84	1.17	nd	0.12	nd	3.89	0.54	1.45	1791	
	Lung	1.43	0.70	0.12	nd	0.08	nd	9.77	1.09	2.45	17	
	Stomach	11.83	5.25	0.52	nd	0.04	nd	4.65	1.42	3.39	nd	
Lamb **	Heart	7.45	3.09	0.71	nd	0.09	nd	6.83	0.03	2.92	590	[117]
	Liver	5.17	2.87	0.48	nd	0.10	nd	8.75	0.81	2.72	152	
	Lung	1.51	0.79	0.11	nd	0.07	nd	8.06	1.10	2.29	12	
	Tongue	8.10	7.57	0.96	nd	0.12	nd	7.17	0.72	1.62	809	
	Stomach	9.99	4.51	0.46	nd	0.05	nd	4.41	1.83	3.17	nd	
Pig *	Heart	40.47	30.06	29.47	0.74	0.77	nd	35.97	nd	nd	nd	[20]
	Liver	43.87	15.73	40.40	0.36	0.92	nd	8.57	nd	nd	nd	
	Lung	49.49	28.65	21.86	0.58	0.44	nd	63.55	nd	nd	nd	
	Spleen	48.85	20.99	30.16	0.43	0.62	nd	90.62	nd	nd	nd	
	Stomach	43.15	38.61	18.24	0.90	0.43	nd	40.86	nd	nd	nd	
Pork *	Liver	48.07	20.37	31.56	0.43	0.66	nd	22.05	0.41	1.59	nd	[58]
	Heart	48.41	40.66	10.51	0.84	0.22	nd	18.13	0.64	1.78	nd	
	Kidney	45.46	43.50	11.04	0.96	0.24	nd	17.59	0.57	1.59	nd	
	Tongue	40.22	48.01	11.78	1.20	0.29	nd	12.31	0.53	1.25	nd	
	Lungs	54.32	26.09	19.60	0.48	0.36	nd	32.22	1.00	2.26	nd	
Wild board *	Liver	46.65	34.76	18.59	0.75	0.40	nd	49.71	0.42	1.62	nd	[58]
	Heart	40.09	36.20	25.60	0.90	0.64	nd	39.05	0.49	1.38	nd	
	Kidney	43.85	34.32	21.81	0.78	0.50	nd	30.52	0.62	1.68	nd	
	Tongue	37.04	51.27	11.59	1.39	0.31	nd	15.28	0.49	1.10	nd	
	Lungs	52.42	39.06	8.52	0.75	0.16	nd	22.25	0.94	2.25	nd	
Red deer *	Liver	44.61	13.95	38.56	0.31	0.87	3.31	1.55	0.32	0.54	nd	[87]
	Heart	29.15	11.34	41.68	0.39	1.51	4.68	3.80	0.28	0.58	nd	
	Kidney	33.57	16.95	42.52	0.50	1.27	3.76	3.16	0.27	0.56	nd	
Emu *	Liver	42.38	27.80	29.82	0.66	0.70	nd	5.40	nd	nd	547.83	[37]
	Heart	37.65	28.53	33.82	0.76	0.90	nd	28.15	nd	nd	159.60	
	Gizzard	38.64	33.68	27.68	0.87	0.96	nd	19.06	nd	nd	332.84	

* Content in % of all fatty acids; ** grams (g) of each individual fatty acid per 100 g edible portion of the sample; nd—no data; SFA—saturated fatty acid; MUFA—monounsaturated fatty acid; PUFA—polyunsaturated fatty acid; h/H—ratio between hypocholesterolemic and hypercholesterolemic fatty acids; AI—atherogenic index; TI—thrombogenic index.

**Table 5:** Content of some vitamins in edible offal.

Vitamin	Animal	Edible Offal	Content	References
Retinol; vitamin A (µg RE/100 g)	Pig	Heart	2.56	[20]
		Liver	57.41	
		Lung	13.37	
		Stomach	17.43	
Thiamine; vitamin B1 (mg/100 g)	Pig	Heart	0.16	[20]
		Liver	0.13	
		Lung	0.11	
		Stomach	0.12	
Niacin; vitamin B3 (mg/100 g)	Pig	Heart	30.96	[20]
		Liver	28.12	
		Lung	0.49	
		Stomach	0.39	
Pantothenic acid, vitamin B5 (mg/100 g)	Pig	Heart	2.95	[20]
		Liver	3.05	
		Lung	3.69	
		Stomach	1.59	
Folates; vitamin B9; (µg/100 g fresh weight)	Cattle	Liver	296–1310	[180,181,182,183,184,185,186,187]
	Pig	Liver	110–1470	[180,182,183,184]
	Chicken	Liver	588–2700	[180,183,184,187]
	Turkey	Liver	677–1137	[180,187]
Cobalamin; vitamin B12 (ng/g)	Cattle	Liver	54.1–78.2	[188,189]
Cholecalciferol; vitamin D3 (µg/kg)	Cattle	Liver	<0.5–14.2 (0.7–7.7) *	[190,191,192,193,194,195,196]
	Cattle	Kidney	1.3–27.1 (1.6–9.8) *	[190,191,194,195,196]
	Pig	Liver	4.0–12.5 (4.4) *	[193,197]
	Chicken	Liver	2.0	[197]

* 25-hydroxychole-calciferol, calcidiol 25(OH)D (µg/kg).

**Table 6:** Content of some microelements (μg/g) and macroelements (mg/g) in edible offal.

Animal	Edible Offal	Microelements					Macroelements				References
		Fe	Zn	Mn	Cu	Se	Na	K	Ca	Mg	
Cattle	Liver	29.3–119.5	29.5–57.0	2.9–5.8	26.8–96.6	39.0–43.2	0.68–0.75	2.81–3.12	0.05–0.06	0.18–0.23	[8,9,14,45,68,231]
	Heart	29.3–101.6	15.3–17.0	2.9	2.7–4.0	21	0.77–0.97	2.69–2.85	0.05–0.08	0.20–0.24	
	Kidney	23.7–279.5	19.5–47.8	1.4–10.0	4.1–4.3	139.0	1.30–1.85	2.56–2.63	0.07–0.13	0.17–0.20	
	Tongue	14.8–21.5	23.2–53.2	0.2–1.0	0.7	12.0	0.69–0.73	2.78–3.15	0.06–0.43	0.16–0.23	
	Lung	201.4	nd	nd	nd	nd	nd	nd	nd	nd	
	Brain	25.5	10.2	0.4	2.0	7.55	1.26	2.74	nd	0.13	
Calves	Liver	29.3–64.0	50.5–120.0	2.6–3.5	26.8–117.7	22.6	0.68–0.75	3.06–3.10	0.03–0.05	0.20–0.23	[9,14]
	Heart	26.4–42.5	12.7–32.8	3.5–4.7	3.5–3.9	32.0	0.69–0.75	2.87–7.61	0.03–0.05	0.18–0.28	
	Kidney	23.5–34.0	19.5–34.0	0.7–1.9	3.4–5.0	80.0	1.53–1.75	2.32–2.72	0.05–0.10	0.16–0.20	
	Tongue	17.8–27.0	26.5–52.4	0.3–3.4	1.5–1.7	6.0	0.80–0.82	2.67–2.70	0.04–0.07	0.15–0.22	
	Brain	21.5	11.5	0.4	2.3	10.0	1.25	3.12	0.01	0.14	
Sheep	Livers	80.1–153.0	14.0–40.2	2.8	11.8–179.6	0.12–0.33	0.63	3.34	0.06–0.09	0.12–0.27	[8,232]
	Hearts	34.7	17.1	0.3	nd	nd	1.09	2.56	0.08	0.23	
	Kidneys	29.7–64.1	9.4–18.8	0.9	1.8–14.8	0.85–1.42	1.50	2.49	0.09–0.13	0.13–0.20	
	Tongues	15.2	16.5	0.1	nd	nd	1.02	2.18	0.07	0.20	
	Lungs	77.6	17.0	0.1	nd	nd	1.49	2.85	0.08	0.17	
	Spleen	974.0	26.9	0.2	nd	nd	1.06	4.64	0.07	0.25	
	Stomachs	41.0	15.7	7.8	nd	nd	0.53	1.34	0.27	0.16	
	Intestines	12.9	9.2	0.4	nd	nd	0.40	0.88	0.11	0.15	
Lamb	Liver	51.5–72.5	30.2–45.5	1.5–1.8	68.8	82	0.67–0.71	3.10–3.12	0.06–0.07	0.21–0.19	[8,9]
	Heart	39.3–40.6	17.7–18.3	0.0–0.5	3.5	33	0.89–1.18	2.80–3.16	0.05–0.06	0.17–0.23	
	Kidney	34.2–63.5	20.6–22.5	0.9–1.2	4.6	125	1.55	2.69–2.75	0.06–0.13	0.17–0.18	
	Tongue	17.7–26.0	19.9–23.5	0.4–0.5	2.0	15.0	0.75–1.12	2.57–2.98	0.07–0.09	0.21–0.24	
	Lung	158.0	17.7	0.2	nd	nd	1.66	2.52	0.07	0.16	
	Spleen	197.0	24.1	0.2	nd	nd	0.95	4.03	0.04	0.20	
	Stomachs	22.7	14.8	0.5	nd	nd	0.58	1.29	0.12	0.12	
	Intestines	13.7	10.0	0.8	nd	nd	0.43	0.94	0.09	0.11	
	Brain	16.5	11.5	0.5	2.5	9.0	1.15	2.95	0.09	0.12	
Pig	Liver	0.5–1444.8	1.9–98.2	0.22–3.5	0.2–44	0.01–80	0.08–1.27	0.10–3.59	0.008–0.20	0.005–0.29	[20,24,33,58,69,118,119,233,234,235,236]
	Heart	2.6–68.1	0.3–28.3	0.4	0.1–4.1	0.01	0.03–1.33	0.05–2.93	0.002–0.09	0.003–0.25	
	Kidney	0.1–93.3	1.7–37.4	0.7–2.3	0.6–9.5	0.06	0.10–1.57	0.08–2.81	0.004–0.14	0.003–0.26	
	Tongue	2.4–29.8	19.9–24.1	0.4–0.8	2.0–2.6	nd	0.72–0.92	2.46–2.72	0.10–0.13	0.17–0.19	
	Lung	63.0–89.6	1.5–20.9	0.2–0.4	1.2–1.9	nd	0.61–1.58	1.89–2.51	0.08–0.21	0.12–0.15	
	Spleen	9.6–274.9	0.7–33.2	0.4–0.5	0.04–2.1	nd	0.12–0.95	0.06–4.64	0.006–0.07	0.003–0.21	
	Stomach	11.1	1.8	0.8	1.4	nd	1.21	1.39	0.13	0.18	
	Small intestine	2.6	1.7	15.6	0.1	0.01	0.03	0.03	0.006	0.004	
	Large intestine	0.9	0.7	2.9	0.01	0.01	0.03	0.03	0.005	0.002	
	Pancreas	38.0	3.8	2.0	1.6	nd	0.85	3.15	0.22	0.23	
	Brain	2.5–38.2	15.6–39.7	0.5	3.2–4.4	nd	1.23–1.42	2.53–3.88	0.008–0.19	0.10–0.15	
Wild boar	Liver	300.2	50.1	3.0	4.0	nd	0.74	2.90	0.16	0.18	[58]
	Heart	51.1	17.5	0.5	10.1	nd	0.63	2.71	0.05	0.23	
	Kidneys	137.4	22.5	1.1	6.7	nd	1.00	1.99	0.09	0.24	
	Tongue	25.9	23.5	0.9	2.0	nd	0.83	2.43	0.26	0.17	
	Lungs	68.5	18.3	0.3	0.8	nd	0.88	2.54	0.12	0.15	
Emu	Liver	2880.9	41.5	0.1	4.4	nd	0.74	3.38	0.05	0.20	[37]
	Heart	59.7	34.6	0.03	3.9	nd	0.81	3.28	0.05	0.22	
	Gizzard	11.8	31.4	0.02	0.6	nd	0.81	3.73	0.05	0.16	
Chicken	Liver	79.3	29.9		3.7	nd	nd	nd	nd	nd	[34]

nd—no data.

## Data Availability

Data are contained within the article. The data used to support the findings of this study can be made available by the corresponding author upon request.
